# Examination of cord blood at birth in women with SARS-CoV-2 exposure and/or vaccination during pregnancy and relationship to fetal complete blood count, cortisol, ferritin, vitamin D, and CRP

**DOI:** 10.3389/fped.2023.1092561

**Published:** 2023-03-16

**Authors:** Eric Mendenhall, Mary Beth Hogan, Matthew Nudelman, Deborah L. Preston, Hayley Weese, Garrett Muckleroy, Jordan Needens, Katherine Addicott, Jessica Dailey Haas, Ashlee Roybal, Dustin Miller, Jesse Cottrell, Cynthia Massey, Balaji Govindaswami

**Affiliations:** ^1^ Department of Pediatrics, Marshall University, Joan C Edwards School of Medicine, Huntington, WV, United States; ^2^ Department of Obstetrics and Gynecology, Marshall University, Joan C Edwards School of Medicine, Huntington, WV, United States; ^3^ Department of Neonatology, Hoops Family Children’s Hospital at Cabell Huntington Hospital, Huntington, WV, United States

**Keywords:** SARS-CoV-2, vaccines, cord blood, acute phase reactants, IL-6, cortisol, ferritin, vitamin D

## Abstract

**Background:**

SARS-CoV-2 is known to manifest a robust innate immune response. However, little is known about inflammatory influences from maternal SARS-CoV-2 infection or maternal mRNA vaccination upon the fetus. In addition, it is unknown if Vitamin D deficiency influences fetal homeostasis or if an anti-inflammatory mechanism to the development of possible innate cytokines or acute phase reactants by the maternal/fetal dyad, in the form of cortisol elevations, occur. In addition, effects on Complete Blood Count (CBC) are not known.

**Objective:**

To evaluate the neonatal acute phase reactants and anti-inflammatory responses after maternal SARS-CoV-2 disease or mRNA vaccination.

**Methods:**

Samples and medical records reviews from mother/baby dyads (*n* = 97) were collected consecutively, and were categorized into 4 groups; no SARS-CoV-2 or vaccination exposure (Control), Vaccinated mothers, maternal SARS-CoV-2 disease positive/IgG titer positive fetal blood, and maternal SARS-CoV-2 positive/IgG titer negative fetal blood. SARS-CoV-2 IgG/IgM/IgA titers, CBC, CRP, ferritin, cortisol, and Vitamin D were obtained to examine the possible development of an innate immune response and possible anti-inflammatory response. Student's *t*-test, Wilcoxon rank-sum, and Chi-squared with Bonferroni corrections were used to compare groups. Multiple imputations were performed for missing data.

**Results:**

Cortisol was higher in babies of both mothers who were vaccinated (*p* = 0.001) and SARS-CoV-2 positive/IgG positive (*p* = 0.009) as compared to the control group suggesting an attempt to maintain homeostasis in these groups. Measurements of ferritin, CRP, and vitamin D did not reach statistical significance. CBC showed no variation, except for the mean platelet volume (MPV), which was elevated in babies whose mothers were vaccinated (*p* = 0.003) and SARS-CoV-2 positive/IgG positive (*p* = 0.007) as compared to the control group.

**Conclusion:**

Acute phase reactant elevations were not noted in our neonates. Vitamin D levels were unchanged from homeostatic levels. Cord blood at birth, showed Cortisol and MPV higher in vaccinated and SARS-CoV-2 IgG positive mother/baby dyads as compared to the Control group, indicating that possible anti-inflammatory response was generated. The implication of possible inflammatory events and subsequent cortisol and/or MPV elevation effects upon the fetus after SARS-CoV-2 disease or vaccination is unknown and merits further investigation.

## 1. Introduction

Current maternal-fetal research into SARS-CoV-2 (COVID-19) disease has centered upon the presence or absence of vertical disease transmission and passive transfer of maternal IgG in both naturally infected and vaccinated mothers (1). Vertical transmission of COVID-19 has been found to be rare (1, 2). This is thought to be related to the significantly low number of ACE2 viral binding receptors in the placenta (1, 3). To date, the innate immune system has been determined to be a significant part of the human response to SARS-CoV2 infection. It is known that SARS-CoV-2 is a highly inflammatory disease with significant elevations in innate cytokines such as IL-1, TNFα, and IL-6, all released by macrophages following induction of the complement cascade (4–6). These cytokines are associated with acute phase reactant development of mediators such as ferritin and CRP, which are released from the liver (6). Elevations in acute phase reactants have been noted in adults and children with severe COVID-19 infection (7, 8). Recent work has focused on the effects of the maternal innate immune response to COVID-19 and the placental response to maternal infection (9).

Protection from COVID-19 infection is an important part of prenatal care. Neonates must rely on both innate immune responses such as cytokine interferon type 1, which has anti-viral properties, the success of maternal defeat of the infection, and maternally acquired transplacental antibodies for protection from disease (10). Multiple studies conclude maternal immunization results in higher, longer-lasting SARS-CoV-2 IgG levels in neonates (11–13). Additionally, it has been shown that maternal immunization also yields immunoglobulin protection in breast milk, specifically IgA (12, 13). This research has given obstetric providers the evidence to further encourage their patients to obtain COVID-19 immunizations as well as remain up to date with boosters (14). During the pandemic, the mRNA vaccines demonstrated protection against the spike protein of COVID-19 (15). In contrast, native disease will generate IgG antibodies to both the spike protein and the nucleocapsid of the virus (15). Passive transfer of maternally derived COVID-19 specific IgG antibody begins 2 weeks after vaccination (13). COVID-19-specific IgG transference from native COVID-19 is more time-dependent, with the highest neonatal titers developed during second-trimester infections as compared to more recent infection during the third trimester (12, 16). The mRNA vaccine mechanism has been studied and generates a facilitated immune response by complement cascade activation acting as the adjuvant to the vaccine (17). Initiation of the complement cascade by either infection or vaccination may have implications on downstream markers of inflammation and the generation of acute phase reactants (18).

With the activation of inflammation and stress, it is likely the mother may mount an anti-inflammatory response to resolve these influences during both infection and vaccination. The neonate also possesses several hormones to affect immune homeostasis if influenced by infection or maternal immune responses. Vitamin D and cortisol are the predominate hormones helping to maintain neonatal anti-inflammatory stance and reestablishing homeostasis. Vitamin D is frequently placental in origin, and cortisol levels in the mother are known to be partially placentally transferred to the fetus (19–21). In addition, stressors of either the mother, neonatal infection, or the birthing process itself may also cause the neonate to generate cortisol on its own (22, 23). This suggests that the bi-directional effect within the maternal/fetal dyad on cortisol has a vital role in the maintenance of neonatal homeostasis.

We hypothesize that innate immune response in both vaccination and COVID-19 disease may increase neonatal exposure to innate cytokines, yielding an inflammatory response by generation of acute phase reactants with associated homeostatic generation of anti-inflammatory elevation of cortisol and Vitamin D levels. In this study, we will assess this hypothesis by evaluating cord blood acute phase reactants ferritin and CRP and any inflammatory influence on the CBC. In addition, we will test whether a corresponding compensatory elevation in cord blood anti-inflammatory hormones of cortisol and Vitamin D in the setting of maternal COVID-19 disease or post-immunization is found.

## 2. Methods

This prospective cohort study sought to collect cord blood samples with either documented maternal SARS-CoV-2 exposure and/or vaccination in pregnancy or mothers who lacked prior SARS-CoV-2 exposure and/or vaccination in pregnancy, however only 97 were found to meet our inclusion criteria during our period of sample collection. Samples were collected consecutively, at Cabell Huntington Hospital's labor and delivery department in Huntington, West Virginia, United States, followed by medical chart reviews of the mother and neonate charts to extract all pertinent data. Exclusion criteria were mothers who were transferred from an outside facility, infants born with congenital defects or disease, any mother/infant dyad who was missing critical information for our dataset, and any mother/infant dyad whose placenta didn't yield enough cord blood for testing. Additional inclusion criteria included neonates delivered after 32 weeks gestational age to ensure transference of immunoglobulins. Dyads that failed screening for technical reasons (gestational age less than 32 weeks) or arrival of the research team in an insufficient time to collect blood due to expected clotting of fetal vessels were not recorded. One-hundred-six samples were collected, and the resulting 97 samples from the cohort of mother/baby dyads were divided into four groups; Control: SARS-CoV-2 negative mothers with no history of vaccination and SARS-CoV-2 IgG negative fetal blood; Vaccinated: vaccinated mothers/IgG positive fetal blood. Unvaccinated/SARS-CoV-2(+)/IgG(+): non-vaccinated SARS-CoV-2 positive/IgG positive fetal blood; Unvaccinated/SARS-CoV-2(+)/IgG(−): non-vaccinated SARS-CoV-2 positive/IgG negative fetal blood. Maternal and neonatal demographics, including birthweights and clinical variables were obtained from a medical records review. A minimum of 6 cc umbilical cord blood was obtained in the first few (typically <10) minutes after placental delivery. A portion of serum from the fetal blood was sent to Cincinnati Children's Hospital Nephrology Lab, Cincinnati, Ohio, United States, for analysis of SARS-CoV-2 specific IgG, IgM, and IgA antibody titers. SARS-CoV-2 specific IgA was obtained to exclude the possibility of maternal blood contamination, as immunologically, the neonate cannot generate IgA immunoglobulin until 2–3 weeks after birth; at the same time, these IgA levels are amply found in the maternal bloodstream (24, 25). CBC, CRP, ferritin, cortisol, and Vitamin D were measured in the cord blood at Cabell Huntington Hospital. Informed consent was not required for this study as cord blood samples were obtained by forfeited placental/cord blood specimens, and maternal/fetal demographics were obtained by medical chart review and de-identified. This research study was approved by the Marshall University Medical Internal Review Board (IRB), IRB# 1726140, prior to its initiation.

### 2.1. Statistical analysis

Descriptive statistics were used to characterize infant and maternal metrics. Mean (standard deviation), and median (25th, 75th interquartile) were used for parametric and non-parametric data, respectively. The Modified Levene's test was used to compare the variance of continuous data between study groups. Student's *t*-test and Welch's *t*-test were used for comparing parametric continuous data between study groups. The Wilcoxon rank-sum test was used to compare non-parametric continuous data between study groups. Pearson's chi-squared test and Fisher's exact test were used for comparing proportional differences in categorical data between study groups. Bonferroni correction was conservatively applied for all multiple comparisons; hence, we considered statistical significance to be a *p*-value < 0.0167. Multiple imputations, using univariate interval regression models, were used for missing CBC data that were missing completely at random (MCAR). Data were evaluated for any gross deviations from statistical testing assumptions.

## 3. Results

A total of 106 cord blood samples were collected from May 2021 to August 2022 after placental delivery. Nine samples were excluded due to missing data, which prevented them from being assigned to a study group. The final study population included 97 samples that were divided into four groups based on maternal SARS-CoV-2 exposure/vaccination and the presence or absence of neonatal SARS-CoV-2 specific IgG.

All vaccinated mothers received mRNA vaccine as they were the only regionally available vaccines during the period of study collection. Vaccination and SARS-CoV-2 exposure data are included in Table 1. Differences in maternal and neonatal demographics were noted between groups (Tables 2, 3). Birth complications were lower in the Unvaccinated/SARS-CoV-2(+)/IgG(+) group compared to the Control group (20% vs. 60%, *p* = 0.005). Respiratory distress syndrome (RDS) was lower in the Unvaccinated/SARS-CoV-2(+)/IgG(+) group compared to the Control group (3% vs. 27%, *p* = 0.024). Neonatal abstinence syndrome (NAS) was lower in the Unvaccinated/SARS-CoV-2(+)/IgG(+) group compared to the Control group (0% vs. 20%, *p* = 0.023). In addition, a greater proportion of vaccinated mothers intended to exclusively breastfeed compared to unvaccinated mothers (Control) (83% vs. 53% *p* = 0.001).

**Table 1:** Maternal vaccination and SARS-CoV2 exposure data stratified by study groups based on SARS-CoV2 exposure/vaccination and presence of neonatal SARS-Cov2 specific IgG, *N* = 97.

	Descriptive statistic	Unvaccinated SARS-CoV-2(−) SARS-CoV-2 IgG(−) [Control]	Vaccinated	Unvaccinated SARS-CoV-2(+)		*P* value		
				SARS-CoV-2 IgG(+)	SARS-CoV-2 IgG(−)	Control vs. Vaccinated	Control vs. Unvaccinated SARS-CoV-2(+) SARS-CoV-2 IgG(+)	Control vs. Unvaccinated SARS-CoV-2(+) SARS-CoV-2 IgG(−)
		*N* = 15	*N* = 32	*N* = 35	*N* = 15			
Tested positive during pregnancy^a^	*n*/*N*, (%)	0/2 (0%)	11/30 (37%)	28/28 (100%)	15/15 (100%)	.534	.002***	.007***
Vaccinated during pregnancy^a^	*n*/*N*, (%)	0/2 (0%)	32/32 (100%)	0/27 (0%)	1/14 (7%)	.002***	1.000	1.000
Fully vaccinated >=2 weeks prior to birth^a^	*n*/*N*, (%)	0/2 (0%)	30/32 (94%)	0/27 (0%)	1/14 (7%)	.011***	1.000	1.000
Vaccine						.002***	<.001***	1.000
Moderna	%	0	25	0	7			
Pfizer	%	0	34	0	0			
Unknown mRNA brand	%	0	41	0	0			
Unvaccinated	%	13	0	77	87			
Unknown if vaccinated	%	87	0	23	7			
Doses Prior to birth						.004***	1.000	1.000
0	%	13	0	77	87			
1	%	0	6	0	0			
2	%	0	91	0	7			
Unknown	%	87	3	23	7			

^a^Missing data imputed for 1 patient.

*** *p* < 0.0167 (Below Bonferroni correction).

**Table 2:** Maternal demographics stratified by study groups based on SARS-CoV2 exposure/vaccination and presence of neonatal SARS-Cov2 specific IgG, *N* = 97.

	Descriptive statistic	Unvaccinated SARS-CoV-2(−) SARS-CoV-2 IgG(−) [Control]	Vaccinated	Unvaccinated SARS-CoV-2(+)		Control vs. Vaccinated	Control vs. Unvaccinated SARS-CoV-2(+) SARS-CoV-2 IgG(+)	Control vs. Unvaccinated SARS-CoV-2(+) SARS-CoV-2 IgG(−)
				SARS-CoV-2 IgG(+)	SARS-CoV-2 IgG(−)			
		*N* = 15	*N* = 32	*N* = 35	*N* = 15			
Rural	%	13	19	29	40	1.000	.304	.215
Smoker	%	20	3	3	33	.089	.075	.682
Second hand smoke exposure^a^	n/N, (%)	3/9 (33%)	1/13 (8%)	3/12 (25%)	5/8 (63%)	.264	1.000	.347
Age at delivery, years	Mean (SD)	29 (6)	29 (5)	27 (5)	29 (6)	.708	.183	.949
Body mass index (BMI) at delivery	Mean (SD)	35 (10)	34 (6)	34 (7)	32 (6)	.527	.645	.236
State of Residence						.697	.524	.052
Kentucky	%	0	6	11	20			
Ohio	%	20	13	20	0			
West Virginia	%	80	81	69	80			
Maternal Race/Ethnicity						1.000	.666	1.000
African American	%	7	6	0	0			
Caucaisian/White	%	93	94	94	100			
Hispanic	%	0	0	3	0			
Unknown	%	0	0	3	0			
Mom's intent to breast feed						.001***	.386	.324
Bottle fed only	%	47	3	29	33			
Breast fed only	%	53	84	66	47			
Supplemental fed (both)	%	0	13	6	20			

^a^Missing data is reflected by a denominator value that is less than the study group *N*.

*** *p* < 0.0167 (Below Bonferroni correction).

**Table 3:** Neonatal demographics stratified by study groups based on SARS-CoV2 exposure/vaccination and presence of neonatal SARS-Cov2 specific IgG, *N* = 97.

	Descriptive statistic	Unvaccinated SARS-CoV-2(−) SARS-CoV-2 IgG(−) [Control]	Vaccinated	Unvaccinated SARS-CoV-2(+)		*P* value		
				SARS-CoV-2 IgG(+)	SARS-CoV-2 IgG(−)	Control vs. Vaccinated	Control vs. Unvaccinated SARS-CoV-2(+) SARS-CoV-2 IgG(+)	Control vs. Unvaccinated SARS-CoV-2(+) SARS-CoV-2 IgG(−)
		*N* = 15	*N* = 32	*N* = 35	*N* = 15			
Male sex	%	47	41	37	60	.696	.529	.715
Gestational age, weeks	Median (IQR)	37 (36, 38)	38 (37, 39)	38 (37, 39)	38 (34, 39)	.151	.075	.782
Birth weight, grams	Mean (SD)	2,898 (466)	3,063 (679)	3,192 (467)	2,866 (826)	.338	.050	.900
Birth length, cm	Mean (SD)	48 (2)	49 (3)	50 (2)	48 (4)	.161	.019*	.965
Birth head circumference, cm	Mean (SD)	33 (1)	33 (2)	34 (1)	33 (2)	.920	.060	.705
Birth body mass index (BMI)	Mean (SD)	12 (1)	12 (3)	13 (1)	12 (2)	.534	.390	.560

* *p* < 0.050.

Differences in infant immunological titers were noted between groups (Table 4). The groups containing Vaccinated and early-in-pregnancy COVID-19 disease (greater than 2 weeks prior to delivery) had IgG titers to SARS-CoV-2 (Table 4). All mothers assigned to the Control group by history also had no evidence of IgG-specific COVID-19 in fetal blood, to exclude the possibility of asymptomatic disease. Most, 27 of the 32 cord blood samples (84%) in the Vaccinated group showed SARS-CoV-2 specific IgG titers above our reference laboratory's upper limit of detection (1:12,800). Eleven of the 32 vaccinated mothers (34%) also had a history of SARS-CoV-2 disease. All 11 of these mothers (100%) showed SARS-CoV-2 specific IgG titers above our upper limit of detection (1:12,800). All mothers who had COVID-19 disease within 2 weeks (Unvaccinated/Sars-CoV2(+)/IgG(−)) prior to delivery did not transfer IgG COVID-19 specific antibody to their neonates (Table 4). Indeterminate (non-titratable) levels of SARS-CoV-2 specific IgM antibody were found in 1 sample from a Vaccinated mother. Vertical transmission and maternal blood contamination are unlikely in this sample, as the mother was vaccinated at least 2 weeks prior to delivery, and SARS-COV-2 specific IgA was undetected (Table 4). Fetal tissues are known to make IgM as early as 10–11 weeks of gestation (26), and this is detectable in fetal blood as early as 13 weeks of gestation (27), but reference nomograms for fetal IgMs suggestive of fetal infection exist for fetuses > 23 weeks and through term (28). Differences in infant inflammatory and anti-inflammatory markers were noted between groups (Table 4). Cord blood ferritin was higher in Vaccinated infants compared to the Control group (182 vs. 118 ng/mL, *p* = 0.033); however, this was not statistically significant after the Bonferroni corrections (Table 4). Percent increase in cortisol was higher in infants of both the Vaccinated (100% increase, *p* = 0.001) and the Unvaccinated/SARS-CoV2(+)/IgG(+) (60% increase, *p* = 0.009) compared to the Control group (Table 4). Quantification of inflammatory measures of CBC and CRP showed normal biological variation (Table 4). No statistically significant findings were noted for the possible inflammatory measures of CBC and CRP except the MPV. While MPV was higher in both the Vaccinated (9.6 vs. 7.9 fL, *p* = 0.002) and Unvaccinated/SARS-CoV2(+)/IgG(+) (9.4 vs. 7.9 fL, *p* = 0.008) compared to the Control group, it did not rise to the level associated with a change due to inflammation (Table 4). No groups were found to have a deficiency in Vitamin D (Table 4).

**Table 4:** Infant inflammatory & anti-inflammatory markers, and immunological titers stratified by study groups based on SARS-CoV2 exposure/vaccination and presence of neonatal SARS-Cov2 specific IgG, *N* = 97.

	Descriptive statistic	Unvaccinated SARS-CoV-2(−) SARS-CoV-2 IgG(−) [Control]	Vaccinated	Unvaccinated SARS-CoV-2(+)		*P* value		
				SARS-CoV-2 IgG(+)	SARS-CoV-2 IgG(−)	Control vs. Vaccinated	Control vs. Unvaccinated SARS-CoV-2(+) SARS-CoV-2 IgG(+)	Control vs. Unvaccinated SARS-CoV-2(+) SARS-CoV-2 IgG(−)
		*N* = 15	*N* = 32	*N* = 35	*N* = 15			
Ferritin, nmol/L	Mean (SD)	118 (82)	182 (97)	177 (223)	182 (103)	.033*	.326	.067
Cortisol, nmol/L	Median (IQR)	5 (2, 7)	10 (6, 14)	8 (5, 13)	8 (1, 19)	.001***	.009***	.118
Vitamin D, nmol/L^a^	Mean (SD)	36 (22)	33 (13)	31 (10)	36 (14)	.467	.200	.948
White blood cells, k/cmm	Mean (SD)	12.2 (5.5)	14.2 (5.8)	13.2 (4.5)	12.4 (6.2)	.264	.495	.929
Red blood cells, m/cmm	Mean (SD)	4.3 (0.6)	4.2 (0.6)	4.2 (0.5)	4.5 (0.6)	.699	.725	.233
Hemoglobin, gm/dL	Mean (SD)	16 (2)	15 (2)	15 (2)	17 (3)	.361	.418	.211
Hematocrit, %	Mean (SD)	48 (8)	47 (7)	47 (7)	52 (8)	.605	.590	.160
Platelets, k/cmm	Mean (SD)	197 (94)	255 (110)	231 (98)	243 (130)	.084	.253	.269
MCV, fL^b^	Mean (SD)	112 (8)	111 (7)	110 (6)	115 (7)	.780	.466	.258
MCH, pg^b^	Mean (SD)	37 (2)	36 (2)	36 (2)	37 (3)	.210	.174	.993
MCHC, gm/dL^b^	Median (IQR)	34 (32, 34)	33 (32, 33)	33 (32, 34)	33 (32, 34)	.115	.290	.911
RDW, %^b^	Mean (SD)	18.0 (1.3)	17.5 (1.6)	17.5 (1.1)	17.7 (1.1)	.299	.166	.572
MPV, fL^b^	Median (IQR)	7.9 (7.6, 8.4)	9.6 (8.5, 10.1)	9.4 (8.1, 10.1)	8.5 (7.7, 9.5)	.003***	.007***	.145
CRP, mg/dl						1.000	1.000	1.000
1.32	%	0	3	0	0			
<0.29	%	100	97	100	100			
COVID Specific IgM						1.000	1.000	1.000
Indeterminate	%	0	3	0	0			
Negative	%	100	97	100	100			
COVID Specific IgA						1.000	1.000	1.000
Negative	%	100	100	100	100			
COVID Specific IgG						<.001***	<.001***	1.000
Indeterminate	%	0	0	23	0			
Negative	%	100	0	0	100			
Positive	%	0	100	77	0			
COVID Specific IgG titer						<.001***	<.001***	1.000
n/a	%	100	0	23	100			
1:800	%	0	0	6	0			
1:1,600	%	0	6	14	0			
1:3,200	%	0	0	20	0			
1:6,400	%	0	9	6	0			
>1:12,800	%	0	84	31	0			

^a^Missing data imputed for 1 patient.

^b^ Missing data imputed for 4 patients.

* *p* < 0.050.

*** *p* < 0.0167 (Below Bonferroni correction).

## 4. Discussion

Inflammation generated by an innate immune system is a critical first response to COVID-19 infection and to the COVID-19 mRNA vaccination and is needed to mount an adequate antibody response to both. To maintain mother/fetal dyad immunologic homeostasis, an anti-inflammatory response can be expected. We sought to determine the influence of inflammatory or anti-inflammatory responses during or following infection of COVID-19 or with mRNA vaccination on fetal acute phase reactant development and subsequent anti-inflammatory response. All vaccinated and known infected mothers transferred anti-COVID IgG antibodies to their fetuses. We observed no statistically significant elevations to markers of inflammation with regard to CRP, Ferritin, and CBC, with the exception of an elevation of the MPV which has an unknown relevance at birth. Anti-inflammatory hormone Vitamin D remained within a normal range for fetal cord blood. We did find statistical significance in elevations of cortisol in the cord blood of vaccinated and previously infected mothers. This novel study is the first to observe that mother/fetal dyads are mounting an anti-inflammatory response *via* cortisol in the presence of COVID mRNA vaccine and disease.

Immunoglobulin transference in our study mirrors that of the current literature (11–13). IgG was transferred to 100% of the vaccinated mothers. SARS-CoV-2 specific IgG titers were significantly elevated in Vaccinated dyads, and there was a wide distribution of SARS-CoV-2 specific IgG titers in our SARS-CoV-2(+)/IgG(+) group. No mother in the Unvaccinated/SARS-CoV-2(+)/IgG(−) group (positive COVID-19 test in the 2 weeks prior to delivery) transferred COVID-19 specific antibody to their infant. This is consistent with what has already been observed in current literature regarding the robust response in immunized individuals in as little as 2 weeks after vaccination but not prior to 2 weeks (12).

COVID-19 disease results in innate cytokine production of TNFα, IL-1, and IL-6 in both adults and children (5). IL-6 and complement products such as C5a drive the production of CRP and ferritin, which are important acute phase reactants (6, 18). These innate cytokines have been associated with hyperferritinemia in severe COVID-19 disease (7). We suspect that high levels of C5a were unlikely to have crossed the placenta to affect fetal acute phase reactant production as determined by the lack of statistical elevations in ferritin and CRP levels in our infants.

IL-6 is an important cytokine that participates in placental health (29). A recent study of mothers with COVID-19 infection who underwent chorionic villous and chorioamniotic membrane biopsy, reviewed innate cytokine production by the fetus (9). Expression of interferon type 1 and IL-6 genes were noted to be low in delivered placental tissue even if the mother was infected with COVID-19 (9). In fact, altered innate cytokine gene expression was still evident in placental biopsies of mothers who were completely recovered from COVID-19 (9). CRP and ferritin were normal in all groups we tested, and the lack of elevation of these in our study suggests it was unlikely that elevated IL-6 maternal cross-placental transfer or fetal production of IL-6 in our dyads was demonstrated (9) as we did not demonstrate an increase in fetal acute phase reactants in our study.

While calcium and inactive Vitamin D metabolites can cross the placenta, it is thought that Vitamin D is largely of placental origin, being synthesized by the placenta and fetal kidney tissues (30). Additionally, Vitamin D's effects on inflammation are to inhibit Th1 proliferation and induce Th2 proliferation, thus assisting in downregulating inflammation which may harm the fetus (31). Maternal deficiencies in pregnancy can contribute to poor placental outcomes, such as issues with implantation in early pregnancy or pre-eclampsia in late pregnancy (19). It is known that healthy maternal populations and non-pregnant females of childbearing age in northern latitudes are typically Vitamin D insufficient (32). In our study, there was little variability observed in our groups, with optimal levels of Vitamin D observed across all 4 groups. This suggests that elevated placental Vitamin D is not required for anti-inflammatory responses to vaccination or maternal COVID-19 disease.

In measuring cortisol, however, we noticed a comparable difference between our infants from vaccinated mothers (Vaccinated), as well as our infants born to mothers who had COVID-19 several weeks prior to delivery, mounting a measurable IgG response (Unvaccinated/SARS-CoV-2(+)/IgG(+)). In these two groups, cortisol was 100% and 60% higher as compared to our Control group respectively. There is evidence that maternal cortisol can cross the placenta and is also thought to be a marker of neonatal stress suggesting that this hormone is critical to maintaining maternal/fetal homeostasis (23). Our elevated cortisol infants had unremarkable newborn nursery courses. The only commonality was their assigned groups of Vaccinated or SARS-CoV2(+)/IgG(+). A smaller percentage of these neonates appeared to have fewer NICU admissions (34% in vaccinated and 14% in SARS-CoV2(+)/IgG(+) as compared to 40% of controls) thus it could be postulated that this stress response potentially contributed to a smoother birth transition excluding the need for a NICU admission. Both groups conceivably experienced inflammation during either vaccination or disease. Demonstrated elevations in cortisol in our study suggest that either mother, fetus, or both inherently tried to achieve homeostasis *via* cortisol to control the damaging effects of inflammation on the fetus. Cortisol is known to decrease NF-*κ*B production of innate cytokines such as IL-1 and IL-6 (33). Our novel study may, in fact, suggest a biologically plausible reason for the lack of innate cytokine production found in biopsied placentas of COVID-19 infected mothers (9).

Our prospective, proof of concept, single-centered study, was small. A larger sample size in the future would enable more robust findings; however, to account for our sample size, we utilized conservative effect estimates calculated using Bonferroni corrections to limit the risk of type I statistical errors. Future studies would be illuminating to include maternal blood sampling to verify cytokine and complement levels and maternal cortisol findings to corroborate our study. Due to the rapid onset of the COVID-19 pandemic, multiple health systems and electronic medical records were not prepared to document patient encounters during the pandemic. As such, our documentation relied on an EMR review of PCP records for testing results or vaccination cards provided by mothers who brought this data to the delivery room. Key strengths of our study were the completeness of our dataset and requiring limited utilization of imputations for missing data. Our novel study demonstrated a statistically significant increase in cortisol which is needed to control neonatal inflammation, but acute phase reactant elevations were not noted. As such, our study may not have accounted for early timing to detect very early innate immune responses and subsequent production of ferritin or CRP yet yielded the downstream anti-inflammatory result of elevated neonatal cortisol levels. In addition, future studies of mothers with long COVID-19 might determine if the development of heightened cortisol levels is due to smoldering inflammation.

We hypothesized in this novel study that either COVID-19 disease or the mRNA vaccine might have inflammatory effects on fetal indices of inflammation such as CBC, ferritin, and CRP. While the CBC showed no variation, but for MPV, the clinical implications of MPV variation at birth are not known. In this novel study attempting to define inflammatory and anti-inflammatory changes notable to a fetus after maternal COVID disease or vaccination, we found that cortisol levels were elevated in these dyads. This finding is important because cortisol may be an important anti-inflammatory response to the innate immune system activation associated with COVID-19 disease or the mRNA vaccine. In addition, a cortisol elevation may provide a hypothesis for previous research determining the lack of fetal gene expression of innate cytokines during maternal COVID-19 infection. Future studies of both maternal and fetal cytokine and complement responses to disease and mRNA vaccination will help elucidate how mother/infant dyads achieve homeostasis in the face of inflammatory challenges.

## Data Availability

The original contributions presented in the study are included in the article/Supplementary Material, further inquiries can be directed to the corresponding author.
